# Membranes with Intrinsic Micro-Porosity: Structure, Solubility, and Applications

**DOI:** 10.3390/membranes9010003

**Published:** 2018-12-26

**Authors:** Haoli Zhou, Wanqin Jin

**Affiliations:** State Key Laboratory of Materials-Oriented Chemical Engineering, Jiangsu National Synergetic Innovation Center for Advanced Materials, College of Chemical Engineering, Nanjing Tech University, 5 Xinmofan Road, Nanjing 210009, China

**Keywords:** microporous polymer membrane, structure, solubility, separation performance

## Abstract

Microporous polymer membranes have been widely studied because of their excellent separation performance. Among them, polymers of intrinsic micro-porosity (PIMs) have been regarded as a potential next-generation membrane material for their ultra-permeable characteristics and their solution-processing ability. Therefore, many reviews have been reported on gas separation and monomers for the preparation of PIMs. This review aims to provide an overview of the structure-solubility property. Different structures such as non-network and network macromolecular structure made of different monomers have been reviewed. Then their solubility with different structures and different separation applications such as nanofiltration, pervaporation, and gas/vapor separation are summarized. Lastly, we also provide our perspectives on the challenges and future directions of the microporous polymer membrane for the structure-property relationship, anti-physical aging, and more.

## 1. Introduction

Membrane technology as a cost-effective and environmentally–friendly process has been given greater attention [1]. Great progress has been made during its development progress, in particular the industrialization of certain processes [2,3,4]. For example, since the first successful industrial application for the membrane-based gas separation by Monsanto in 1979–1980 [4], gas separation membranes have found many applications in various processes including the separation of C_2_-C_4_ olefins from nitrogen in petrochemical plants, of nitrogen from air, of volatile organic compounds from air, and more [2,3,4]. The steady growth of membrane-based gas separation systems has generated sales of about $1 billion [4].

Due to the wide applications, extensive research has been conducted in search of better membrane materials [5,6,7,8,9,10,11,12]. Thousands of new materials have been synthesized and evaluated. Unfortunately, most of these new materials did not reach commercial use because of some common problems such as plasticization, physical aging phenomena etc. [3]. For example, the first generally accepted microporous polymer membrane material, poly[1-(trimethylsilyl)-1-propyne (PTMSP), which was discovered by the research group of Higashimura and Masuda [13], exhibits a very high oxygen permeability coefficient of about 6000 barrer and moderate selectivity for O_2_/N_2_ separation at 25 °C, while the corresponding rubber polymer membrane polydimethylsiloxane (PDMS) exhibits an oxygen permeability of only about 500 barrer [14]. Therefore, PTMSP is regarded as one of the most permeable polymers known [15,16]. The emergence of this glassy polymer membrane changed the traditional idea that the permeabilities of glassy polymer membranes are lower than those of rubber polymer membranes. Unfortunately, due to its poor chemical resistance against hydrocarbon liquids [17] and much faster physical aging in comparison to other glassy polymers [3], PTMSP membranes only experienced short popularity. Microporous polymer membranes had not attracted much attention until 2004, when polymers of intrinsic micro-porosity (PIMs) were developed by Budd and McKeown [8,9]. Since then, the microporous polymer membrane has come of age because they show superior transport properties and good selectivity for the separation of gases, vapors, and liquids. Furthermore, its physical aging can be well controlled via a certain modification, although, thus far, it still cannot be completely eliminated [18]. The first commercial application of PIMs already occurred in 2015. However, it involves the detection of organic vapors instead of its use as a separation membrane [19,20]. This successful commercialization nonetheless renewed the hope for the future industrialization of PIMs or microporous polymer membrane. Many papers have been published on optimal PIMs membranes, the development of PIMs membranes with new structures, new applications, and so on [3,5,21]. To date, more than 400 papers have been published on the synthesis, modification, physical properties, and applications. However, to the best of our knowledge, there has not been a review about the comparison of properties between a non-network (linear) and a network structure in microporous polymers, such as solubility and film-forming properties especially when the thickness is less than 100 nm. In addition, most reviews have focused on gas separation, while other separations such as pervaporation have gained less attention even though they also exhibited good performance, while these separations would widen the applicability of the microporous membrane. This article attempts to review different structures in the microporous membrane. Specifically, the non-network and the network structure in microporous polymer membranes with different monomers are reviewed and compared. Physical aging is an important problem in microporous polymer membranes, but this topic has already been investigated and reviewed [3,22,23,24,25]. Therefore, only a brief review on physical aging is given in this paper. Lastly, methods for accurately estimating the specific surface area of the membranes and potential applications beyond gas separation are also briefly reviewed.

## 2. Definition of the Gas Separation Membrane

‘Microporous’ is a term with different meanings in different professional fields. In membrane technology, before extensive research on microporous membranes had been conducted, it was also applied to membranes containing pores larger than 50 nm (macroporous by the International Union of Pure and Applied Chemistry (IUPAC) definition) [26]. For example, membranes with a nominal pore size ranged from 0.05 μm to 0.45 μm and were also named microporous membranes [27,28,29], which may cause misunderstanding or confusion, because this does not align with the IUPAC definition and other fields such as adsorption and catalysis wherein the term microporous refers to pore sizes smaller than 2 nm [30,31]. Many microporous membranes such as PIMs etc. with pore sizes smaller than 2 nm have been developed and intensively studied recently. Therefore, a new name should be given to this kind of membrane in order to differentiate them from the ones with a much larger pore size. While, in 2015, Thommes et al. [32] reported that micropores can be further subdivided into two types: narrow micro-pores (also named ultra-micro-pores) with an approximate width less than 0.7 nm and wide micro-pores (also named supermicropores). This definition has already been used in a few publications [18,33,34,35,36,37], but it has not been widely adopted. The main disadvantage with the name micropore may be that it does not in itself clearly describe the sizes of the pores in these membranes. It might as well be regarded as the UF (ultrafiltration). A membrane-based gas separation is a process of separating molecules, which have a size on the angstrom scale. Just like nanofiltration, a process rejects molecules that have a size in the order of one nanometer [38]. A suitable name for it is angstrom-filtration using angstr-porous membranes. This occurs in order to completely differentiate them from other so-called microporous membranes such as UF and MF (microfiltration) membranes. When a membrane is used for gas separation, its pore size should be as small as the angstrom scale. For example, the kinetic sizes of common gases such as CO_2_, N_2_ etc. are all smaller than 1 nanometer [39]. Thus, we believe that the angstr-porous membrane is perhaps a more suitable term for the definition of membranes used for gas separation in the future. However, in this review, we continue to use the microporous membrane as it has been reported.

## 3. Design of Microporous Structure in Membranes

### 3.1. Non-Network Macromolecular Structure in Membranes

**Figure 1:**
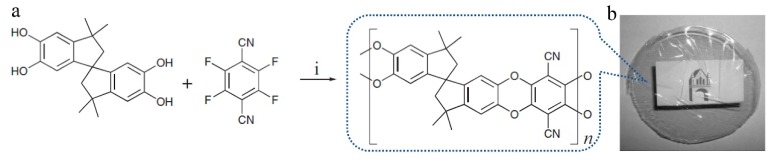
Synthesis of the polymer of intrinsic micro-porosity (i: reagents and conditions: K_2_CO_3_, DMF (*N*,*N*′-dimethylformamide), 50–70 °C). Adapted from Reference [9].

The structure of a membrane material is very important not only because it can affect the physical properties, but also because it affects the performances of a membrane, such as separation performance, adsorption performance, and so on. Hence, in the microporous polymers, two structures known as the non-network macromolecular structure and the network macromolecular structure have been synthesized or discovered in polymers based on different monomers. The former is generally obtained by polymerization of monomers with four reactive sites, while the latter is achieved using monomers with three or six reactive sites. The prototype PIMs first reported by McKeown and Budd [8,9] has been synthesized by an efficient dibenzodioxane-forming reaction between bis-catechol 5,5A,6,6A-tetrahydroxy-3,3,3A,3A-tetramethyl-1,1A-spirobisindane and 2,3,5,6-tetrafluorophthalonitrile to give a non-network polydioxane (Figure 1).

**Figure 2:**
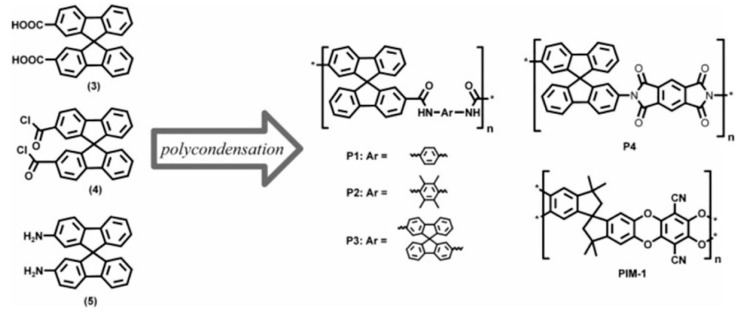
Chemical structures of the spirobifuorene monomers, the resulting poly(amide)s and poly(imide) P1–P4, and the repeating unit of PIM-1. Reproduced from Reference [41].

**Figure 3:**
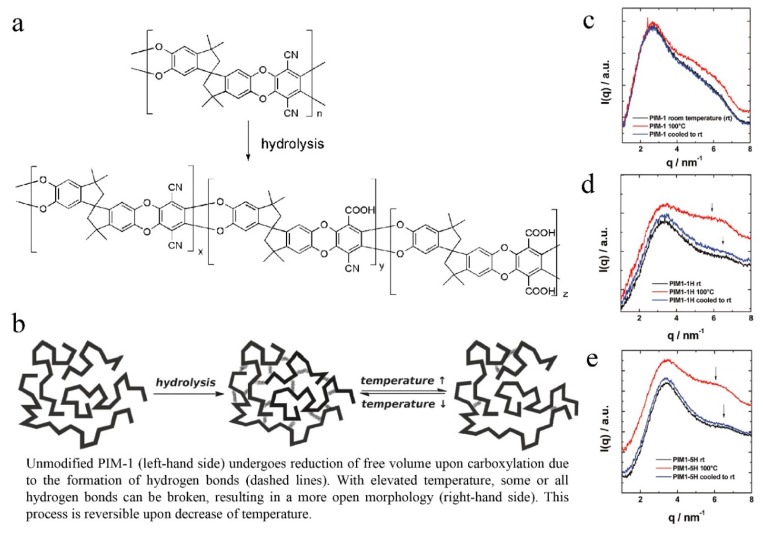
Reaction scheme for hydrolysis of PIM-1 (**a**), representation of the microstructural and hydrogen-bonding changes of PIM1 upon carboxylation (**b**), small-angle x-ray scattering (SAXS) patterns of samples (PIM-1 (**c**), PIM1-1H (**d**), and PIM1-5H (**e**). The membranes were initially measured at room temperature (rt), then at 100 °C, and again after cooling to room temperature. Adapted from Reference [42,43].

This kind of polymer gives a surface area of 850 m^2^/g because their highly rigid and contorted molecular structures cannot fill space efficiently, which results in higher micro-porosity, interconnectedness, and free volume. The advantage of PIMs over other conventional microporous materials is their solution processability, as reported by McKeown [19,40]. Since the development of PIMs, only inherently rigid dibenzodioxan-type polymers have been regarded as having intrinsic micro-porosity until 2007, when the synthesis of aromatic polyamides was carried out, starting from 2,2′-dicarboxy-9,9′spirobifluorene and aromatic polyimide based on 2,2′-diamino-9,9′-spirobifluorene [41], as shown in Figure 2. This report also found an interesting phenomenon that intermolecular interactions such as H-bonding, as observed in the backbones of polyamides and polyimides, could lead to reduced or inaccessible micro-porosity [41,42]. Du et al. [43,44,45] fabricated a carboxylated PIM by partial hydrolysis of the nitrile group of PIM-1 (Figure 3). The advantage of this approach is that the structure of the polymer backbone is unaffected, while the intermolecular interactions, i.e., the number of carboxyl groups throughout the polymer, can be adjusted. Consequently, the selectivity and permeability can be easily tuned by varying the conditions of the hydrolysis reaction. Compared with the PIM-1 membrane, membranes based on carboxylated PIMs with different degrees of hydrolysis had the same thermal and mechanical properties but achieved higher selectivity for different gas pairs such as O_2_/N_2_, CO_2_/N_2_, He/N_2_, and H_2_/N_2_ and the permeability decreased when the degree of carboxylation was increased because of decreases in porosity and free volume. Therefore, if pronounced micro-porosity is to be achieved, bedsides requiring a rigid and kinked backbone as well as a reduction in the number of potential interaction sites (for example, amides) must also be considered [42].

**Figure 4:**
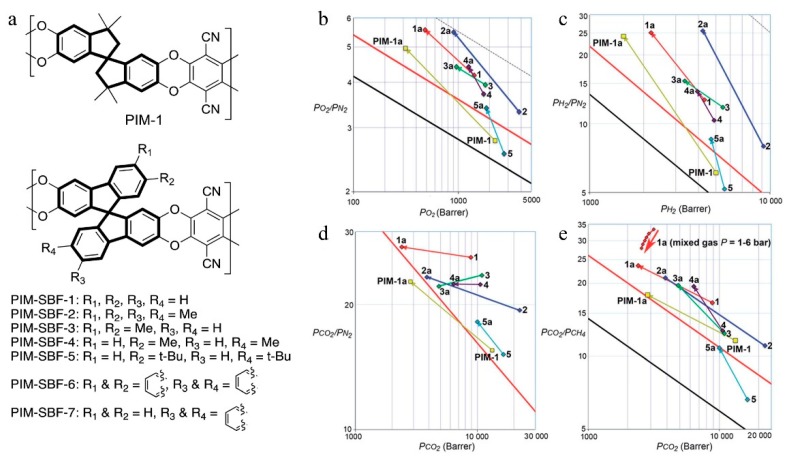
The structures of the spirobisindane-based PIM-1 and the spirobifluorene-based PIM-SBF series (**a**), Robeson plots for the (**b**) O_2_/N_2_, (**c**) H_2_/N_2_, (**d**) CO_2_/N_2_, and (**e**) CO_2_/CH_4_ gas pairs showing the position of the gas permeability data for PIM-SBFs 1–5 and that of PIM-1. Data from films aged for >3.5 years (e.g., 2a) are joined by an arrow to those of the freshly methanol treated films to form aging trend-lines. Upper bounds are represented by black (1991), red (2008), and dotted (2015) lines. Mixed CO_2_/CH_4_ data at pressures of 1–6 bar for the film of PIM-SBF-1 aged for 2088 days are also plotted with a red arrow, which indicates increasing pressure. Adapted from Reference [50].

With the development of microporous polymers, it was found that enhancement of the rigidity of the polymer chains would improve the separation performance of PIM membranes [46,47]. PIM-1 exhibits both flexible and rigid parts of the backbone, according to simulations, where stress-induced distortion of the 1,1′-spirobisindane unit with a large variation in dihedral angles at the spiro atoms of the polymer chain can be observed [48]. Therefore, if the conformational flexibility about the spiro-centers can be restricted to a certain extent, the fabricated membrane will show higher separation performance. Bezzu et al. [49], thus, synthesized a new PIM using a more rigid 9,9′-spirobifluorene (SBF) unit to replace the 1,1′-spirobisindane (SBI) unit in order to improve the stiffness of the polymer chains. Enhanced selectivity for the separation of O_2_/N_2_ was obtained in comparison to PIM-1, without loss of permeability, and their data lie significantly above the 2008 Robeson upper bound, which demonstrates that increased polymer chain rigidity can enhance the selectivity of the membrane. This also identified that the SBF unit is a useful monomer in the synthesis of microporous polymers. Bezzu et al. [50] later used modified SBF units to synthesize a series of PIM-SBF membranes (Figure 4) to investigate the effect of substituents such as methyl, *t*-butyl, and fused benzo groups on the polymer micro-porosity and gas separation performance. Results showed that both the methyl and *t*-butyl substituents enhanced the concentration of micropores and the gas permeability. Specifically, four methyl groups on the SBF units enhanced the concentration of ultra-micro-pores (<0.7 nm). Therefore, PIM-SBF-2 exhibited the highest gas permeability among the four membranes tested, since this pore size is similar to the kinetic diameter of gas molecules and thus more conducive to gas transport. In contrast, *t*-butyl substituents improved the concentration of larger, less selective micropores (>1 nm) because of its slightly larger size. These pores may facilitate the transport of larger gas molecules such as N_2_ and CH_4_. The authors also investigated the long-term aging performance (>3.5 years). Expectedly, better selectivity was obtained for pairs such as O_2_/N_2_, due to the reduced number of micropores resulting from physical aging. The performance of PIM-SBF-2 after aging for 1295 days was very close to the 2015 upper bound for O_2_/N_2_. These studies demonstrate that side groups (size, dimensions, etc.) can greatly affect the micro-porosity and separation performance, and that pore size can be fine-tuned by selecting suitable substituent groups.

**Figure 5:**
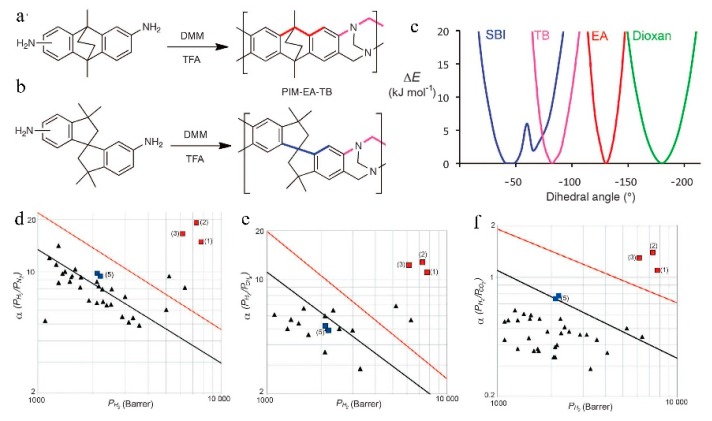
The synthesis and molecular structures of (**a**) PIM-EA-TB, (**b**) PIM-SBI-TB (DMM, dimethoxymethane, TFA, trifluoroacetic acid), (**c**) A plot showing the increase in energy associated with the deviation in the marked dihedral angle within the bridged bicyclic units of EA (red) and TB (purple) as compared with typical components of PIMs, such as the spiro-center of SBI (blue) and the dioxan linking unit (green). The narrower energy wells of the bridged bicyclic units TB and EA demonstrate their greater rigidity. Portions of the Robeson plots relevant to highly permeable polymers for (**d**) H_2_/N_2_, (**e**) H_2_/CH_4_, (**f**) H_2_/CO_2_ gas pairs showing the data for methanol-treated PIM-EA-TB, with data points 1 (solid red square) for a 181 mm film, 2 (solid red square) for a 95 mm film, and 3 (solid red square) for the same 181 mm film after aging for 24 h. The black and red lines represent the 1991 and 2008 upper bounds, respectively. Data points 5 (solid blue squares) are from 157 and 128 mm films of PIM-SBI-TB. Other data points (solid black triangles) represent PIMs and other highly permeable polymers that have been reported since the upper bounds were updated in 2008. Adapted from Reference [51].

Although PIM-1 and PIM-SBF achieve good separation performance for most gas pairs of interest, the spiro-centers and dioxin linking groups in these polymers are still not rigid enough. In order to further increase their separation performance, more rigid monomers should be chosen or synthesized. For this purpose, Carta et al. [51] used Tröger’s base (TB) to synthesize microporous polymers because its bridged bicyclic ring structure is rigid (as confirmed by modeling) and it has a non-linear shape, which could provide the prepared membrane with extreme rigidity and high performance. This was also the first time that the TB unit was used as a component for polymer synthesis. Two kinds of TB-based polymers that are highly soluble in chloroform have been synthesized including PIM-EA-TB (EA, ethanoanthracene) and PIM-SBI-TB (Figure 5). The polymers had apparent BET surface areas of 1028 and 745 m^2^/g for PIM-EA-TB and PIM-SBI-TB, respectively. The higher BET surface area of PIM-EA-TB than those of PIM-SBI-TB and previously reported PIMs can be ascribed to its enhanced micro-porosity because the EA unit also has a bridged bicyclic ring system, which endows it with even higher rigidity than that originating from the TB alone. Unusually, the PIM-EA-TB is more permeable to H_2_ than to other gases. Therefore, its data lie well above the 2008 Robeson upper bound for H_2_/N_2_ (Figure 5) due to the molecular sieving characteristics. With the advantages of the TB unit, Zhuang et al. [52,53,54] also synthesized a series of intrinsically microporous soluble polyimides by incorporating the TB unit into the polymer backbone. These polymers exhibited high fractional free volumes and micro-porosities, which results in significantly higher gas permeabilities in comparison to those of conventional polyimides. Williams et al. [55] employed a rigid methanopentacene (MP) structural unit to form PIM-MP-TB via a polymerization reaction involving the formation of TB linking groups. The higher rigidity of the structures of MP and TB also endowed the PIM-MP-TB with high selectivities for gas pairs such as H_2_/N_2_, which is well above the 2008 Robeson upper bounds. All of these examples provide evidence that molecular rigidity is very important for the intrinsic micro-porosity and size selectivity of polymer membranes.

**Figure 6:**
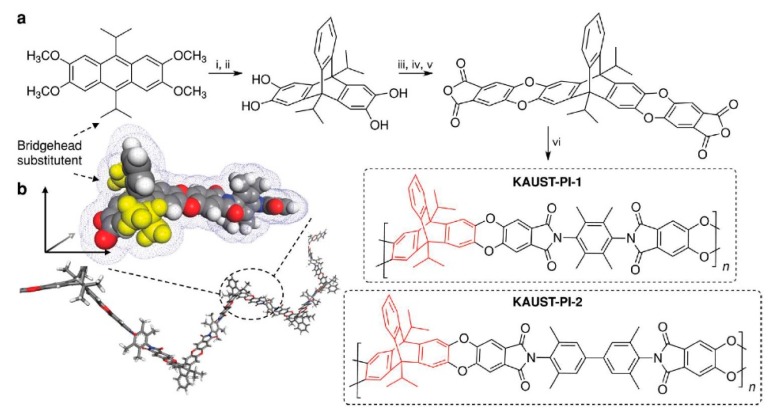
Synthesis of KAUST-PI-1 (**a**). (i) benzene diazonium chloride, (CH_2_)_2_Cl_2_, 80 °C, 4 h. (ii) BBr_3_, CH_2_Cl_2_, 3 h. (iii) 4,5-dichlorophthalonitrile, K_2_CO_3_, *N*,*N*-dimethylformamide (DMF), 80 °C, 10 h. (iv) KOH, C_2_H_5_OH/H_2_O, reflux 10 h. (v) acetic anhydride, reflux 12 h. vi. diamine (TMPD or TMBZ), m-cresol, isoquinoline, 200 °C, 4 h. Geometrically optimized KAUST-PI-1 demonstrating contorted, ribbon-like growth and enhanced three-dimensionality afforded by the 9,10-diisopropyl-triptycene (**b**). Reproduced from Reference [34].

**Figure 7:**
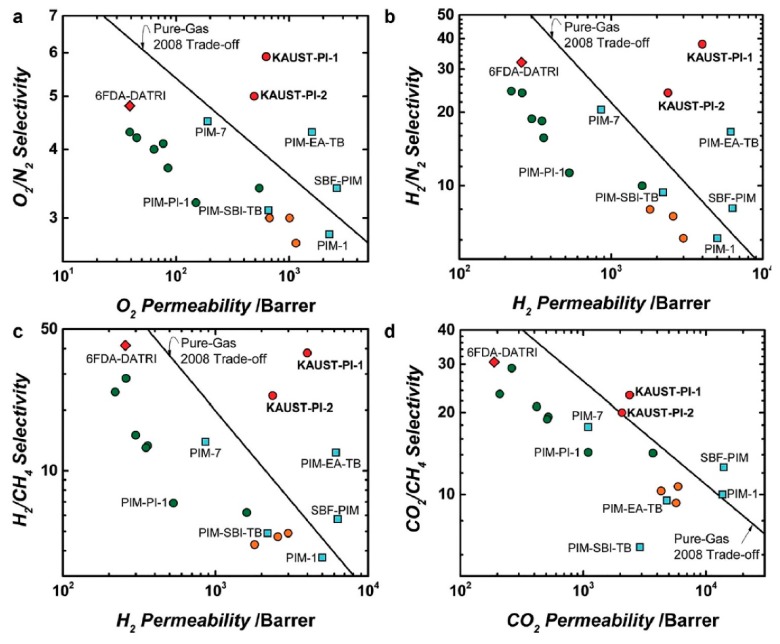
Separation performance for (**a**) O_2_/N_2_, (**b**) H_2_/N_2_, (**c**) H_2_/CH_4_, and (**d**) CO_2_/CH_4_ gas pairs showing data for KAUST-PI-1 and KAUST-PI-2. Symbols represent notable materials from various classes including 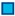 = Ladder PIMs, 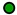 = Extended and 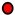 = Non-extended PIM-PIs (Extended PIM-PIs means the reactive anhydride was grafted on the dibenzodioxin units by the introduction of 4,5-dichlorophthalonitrile instead of on the 1,1-spirobisindane unit, while, in the non-extended PIM-PIs, anhydride groups are fused directly to the 1,1-spirobisindane unit [61,62]). Solid lines represent state-of-the-art 2008 permeability/selectivity upper bounds. Reproduced from Reference [34].

Therefore, another important rigid monomer, triptycene, was investigated for synthesis of triptycene-based microporous polymers [18,33,34,56,57,58,59,60]. Triptycene is a unique building block not only because it can be used for the synthesis of high-performance microporous membranes due to its rigid Y-shaped structure with intrinsic internal free volume, but also because it has six active sites on the benzene rings and the 9,10-bridgehead can be used to control a poly-condensation reaction and tune the microporous structure [18]. A triptycene-based PIM-PI (polyimide) type microporous polymer named KAUST-PI (Figure 6) has been synthesized [34], which shows high selectivity and permeability, and easily surpassed the 2008 upper bound limit for the separation of O_2_/N_2_, H_2_/N_2_, H_2_/CH_4_, and CO_2_/CH_4_ (Figure 7). These membranes have also been used to define the 2015 upper bound limits for the separation of O_2_/N_2_ due to its high separation performance [36].

As discussed above, side groups can influence the membrane structure and performance. Therefore, two new monomers known as 9,10-dialkyl (methyl and isopropyl) triptycene-based dianhydride monomers synthesized by Pinnau’s group were used in the synthesis of polyimides in a reaction with 3,3′-dimethylnaphthidine (DMN) via a one-step high-temperature solution polycondensation reaction. Short methyl groups in the bridgehead of the triptycene provided this polyimide with higher BET surface area and greater ultra-micro-porosity, which is consistent with the report that short bridgehead substitutes confer the greatest micro-porosity and high BET surface area [40,50]. This membrane showed high gas separation capability and moderate selectivity for O_2_/N_2_ and H_2_/N_2_ separation, but still surpassed the 2008 upper bound limit. This study demonstrates that bridgehead substituents can be selected to fine-tune the microporous structure, pore size distribution, and, consequently, the separation performance of the membranes.

### 3.2. Network Macromolecular Structure in Membranes

**Figure 8:**
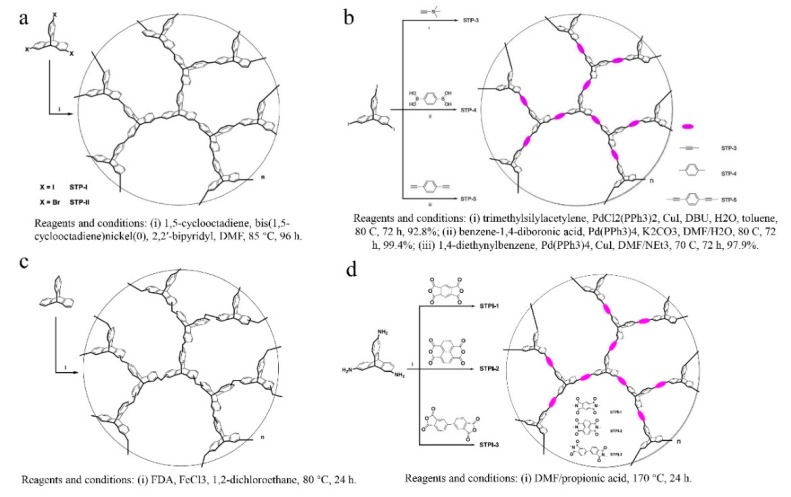
Synthesis of star triptycene-based porous polymers (STPs) (**a**,**b**), triptycene-based hyper-cross-linked polymer sponge (THPS) (**c**), star triptycene-based porous polyimides (STPIs) (**d**). Adapted from References [63,64,65,66].

The following is Figure 8 [63,64,65,66].

**Figure 9:**
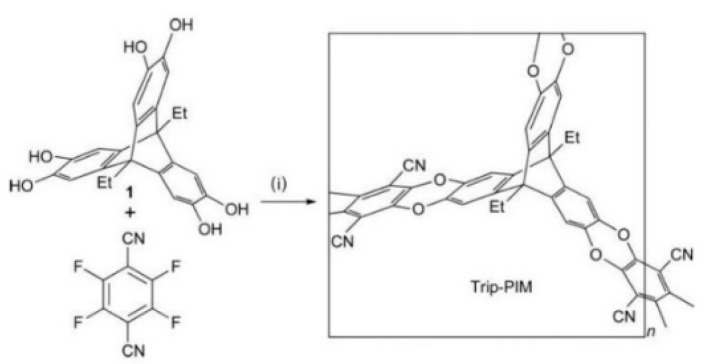
Synthesis of the triptycene-based PIM (Trip-PIM). Reagents and conditions: (i) K_2_CO_3_, DMF, 80 °C. Reproduced from Reference [70].

**Figure 10:**
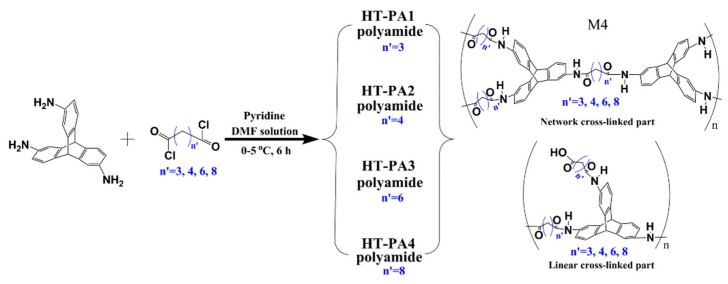
Synthesis of triptycene-based polyamides by solution polymerization. Reproduced from Reference [71].

Based on the above discussion and a recent review [3], it can be concluded that most of the polymer backbones of PIMs consist of non-network macromolecular structure, which is regarded as a major difference from other types of network porous organic polymers such as covalent-organic-frameworks (COFs) that are generally crystalline and insoluble in organic solvents [19]. However, it is anticipated [40,65] that a network microporous polymer prepared using similar monomers along with this kind of materials, which would possess even greater micro-porosity. In order to obtain the network structure in PIMs membranes, monomers with more than three active sites besides a rigid structure, contorted structure, or the spiro center, should be used. To this end, triptycene is supposed to be a suitable candidate. Zhang and Chen’s group [63,64,65,66,67,68,69] thoroughly studied the modification of triptycene and preparation of triptycene-based network microporous polymers (Figure 8). For example, they used 2,6,14-trihalotriptycene as the monomer to synthesize a novel network structure via a triptycene−triptycene coupling reaction. With this method, a BET surface area as high as 1900 m^2^/g can be obtained, which demonstrates the advantage of the network structure. Later, Zhang [65] used 2, 6, 14-triaminotriptycene as the starting material, which react with bifunctional carboxylic acid dianhydrides in order to prepare network polyimides. The results showed excellent adsorption ability in terms of CO_2_ uptake capacity of 14.6 wt% at 273 K, and selectivity in the separation of CO_2_/N_2_ as high as 107. Ghanem et al. [40,70] also focused on the synthesis of network microporous polymers using triptycene as the monomer. Polymerization has been achieved by the reaction between 9,10-diethyl-2,3,6,7,12,13-hexahydroxytriptycene and 2,3,5,6-tetrafluoroterephthalonitrile (Figure 9). A BET surface area of 1064 m^2^/g was obtained, which is higher than that of other PIMs with non-network macromolecular structures. Zhou et al. [71] adopted a new way to synthesize the triptycene-based network microporous polymers (Figure 10) by reacting 2, 6, 14-triaminotriptycene with acyl chlorides, and the resultant polymers exhibited good solvent resistance and anti-swelling properties.

**Figure 11:**
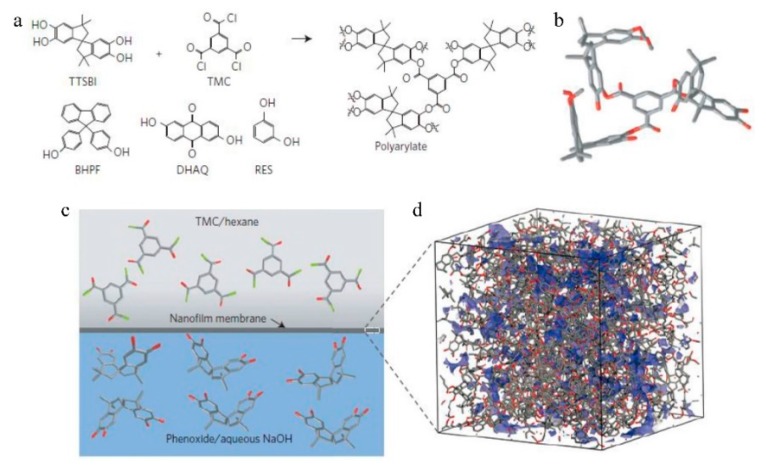
Interfacial synthesis of polyarylate nano-films. (**a**) Synthesis of aromatic polyester (polyarylate) nano-films through interfacial polymerization. The aromatic phenol is dissolved in a dilute sodium hydroxide solution and reacts with trimesoyl chloride (TMC) dissolved in hexane at the hexane/aqueous interface. Four different phenol monomers were used including spiro-structured 5,50,6,60-tetrahydroxy-3,3,30, 30-tetramethylspirobisindane (TTSBI), cardo-structured 9,9-bis(4-hydroxyphenyl)fluorene (BHPF), and planar-structured 2,6-dihydroxyanthraquinone (DHAQ), and 1,3-benzenediol (RES). The cardo-structured and spiro-structured monomers are contorted, rigid monomers. DHAQ and RES are monomers with planar structures. (**b**) Molecular model of a segment of polyarylate network containing spiro-contorted monomers from TTSBI. (**c**) Visualization of the interfacial polymerization between TMC in hexane and the phenoxide of TTSBI in aqueous NaOH solution. (**d**) Three-dimensional view of an amorphous cell containing a spiro-contorted PAR-TTSBI polyarylate network. Blue color: accessible surface at probe radius of 1 Å. Cell size: 46 Å × 46 Å × 46 Å. Reproduced from Reference [39]. 4. Surface area of microporous materials.

Livingston [39] selected a different way to fabricate network microporous polymer membranes by interfacial polymerization with contorted and non-contorted monomers (Figure 11), which achieve a selective layer as thin as 20 nm. The nanofilm prepared with contorted monomers displayed higher micro-porosity and interconnectivity than those fabricated with non-contorted monomers. Permeance to organic solvents of up to two orders of magnitude higher, along with higher adsorption of gases, were also obtained. These examples indicate that, when a polymer with a network structure can be obtained, it may display excellent properties in the aspects of micro-porosity and film-formation.

## 4. BET Surface Area of Microporous Material

McKeown [72] reported that microporous materials are defined as solids with interconnected pores of less than 2 nm and large and accessible surface areas-typically 300–1500 m^2^/g, as measured by gas adsorption. Generally, molecular nitrogen is selected as a probe. The measurement of micro-porosity depends on the interconnected free volume, which has to be open and accessible for the nitrogen molecule probe to detect it [41,72]. This also indicates that, if the probe nitrogen cannot enter the spaces within microporous materials, or if pore deformation during the adsorption process blocks the entrance of nitrogen, the micro-porosity would be underestimated, especially in ultra-microporous samples (pores < 0.7 nm) [41,42]. For example, Livingston [39] prepared network polymers with a pore size of approximately 0.6 nm. When these polymers were conducted in N_2_ adsorption, the BET surface areas were lower than 40 m^2^/g even though the synthesized polyarylate (PAR) polymers showed high solvent permeance and gas permeation. These authors suggested that the low nitrogen uptake resulted from the restricted access of N_2_ molecules within the narrow micropores in the rigid polymer network. A similar observation was also reported by our group [71]. Another report also showed that higher surface area, as tested by argon sorption, could be obtained for COFs [73]. Weber et al. [42] consider this underestimation to be a result of kinetic effects. Therefore, other probe molecules such as CO_2_, which do not suffer from the kinetic effect, may be more suitable for the analysis of microporous materials, especially when the pore size is lower than 0.7 nm. In addition, CO_2_ has already been used successfully for the analysis of microporous polymers [39,71,74,75]. The advantages of CO_2_ sorption include faster measurement times due to the higher kinetic energy and smaller kinetic diameter of CO_2_ [42,74] in comparison to those of N_2_. Data evaluation methods based on a nonlinear density functional theory (NLDFT) or grand-canonical Monte Carlo (GCMC) models can be applied to analyze the adsorption/desorption of CO_2_.

## 5. Solution-Processable Property

**Figure 12:**
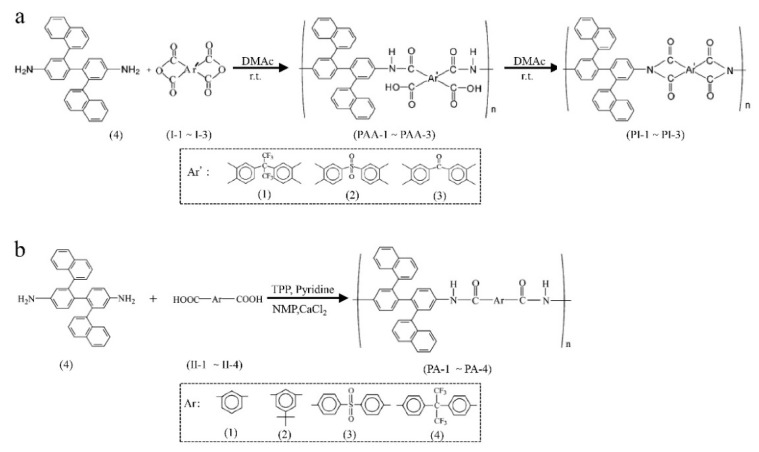
Synthesis of various polyimides (**a**) and polyamides (**b**). Adapted from Reference [82].

**Figure 13:**
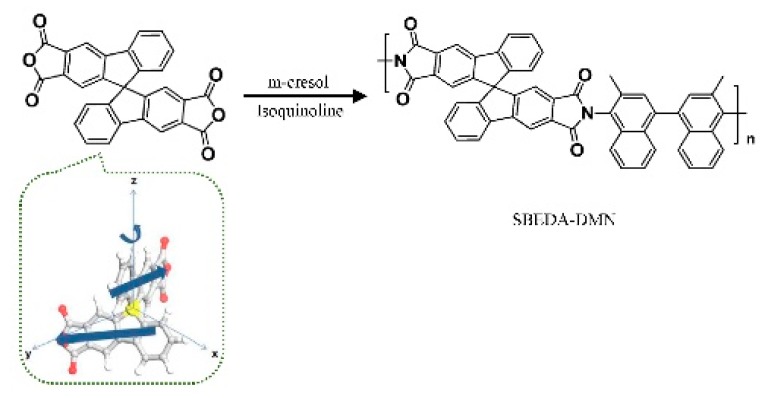
Synthesis of spirobifluorene dianhydride-dimethylnaphthidine (SBFDA-DMN). Adapted from Reference [83].

In the development of polymer membranes, solution-processable property is the key point that determines whether the polymer can be used for further applications, such as the fabrication of membranes using a solution-processing technique, since the solution-coating method is an easy system in the industrialization of polymers [76]. Microporous polymers with solution processability are, therefore, highly desirable for the fabrication of next generation porous membranes. However, most of the microporous materials developed, such as COFs, MOFs (metal-organic framework) etc., are insoluble in common solvents [19,63,73,77,78,79,80]. Therefore, in order to enhance the solubility in common solvents, different methods have been proposed, such as the introduction of bulky lateral substituents, flexible alkyl side chains, or unsymmetric, alicyclic, and kinked structures [81]. Besides these methods, Liaw et al. [82] proposed the incorporation of nonlinear moieties such as bulky and noncoplanar naphthalene groups in the polymer backbones of both polyamides and polyimides to enhance the solubility (Figure 12). Excellent solubility was obtained because the incorporation of naphthalene groups rendered the polymer noncoplanar and also reduced interchain interactions. Pinnau et al. [33,83] used sterically hindered and contorted di-anhydrides such as spirobifluorene di-anhydride (SBFDA) for the synthesis of polyimides, which also showed good solubility in common solvents. This solubility may be a result of their noncoplanar structure (Figure 13).

**Figure 14:**
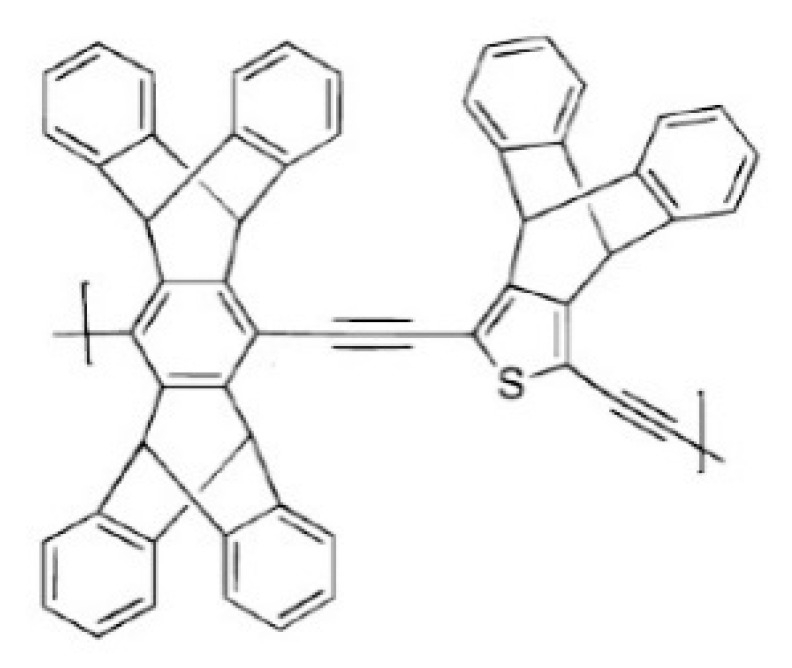
Structural diagram of poly(aryleneethynylene). Reproduced from Reference [84].

Besides the fact that bulky and noncoplanar monomers can enhance the solubility of microporous polymers, triptycene can also increase the solubility of polymers under certain conditions, as reported by Swager’s group [81,84]. Figure 14 shows a polymer, which is highly soluble in dichloromethane, obtained by Sonogashira-Hagihara coupling of a diiodothiophene monomer with diethynylpentiptycene in dichloromethane. This is the first example of a soluble poly(aryleneethynylene) composed entirely of an iptycene monomer unit instead of a flexible side chain. Its solubility appears to derive from the high concentration of iptycene groups and its nonlinearity, where thiophene groups on adjacent repeat units provide a crucial disorder that prevents the polymer from crystallizing. Meanwhile, the inhibition of chain interactions by the iptycene groups in the backbone enhances the solubility of this polymer.

**Figure 15:**
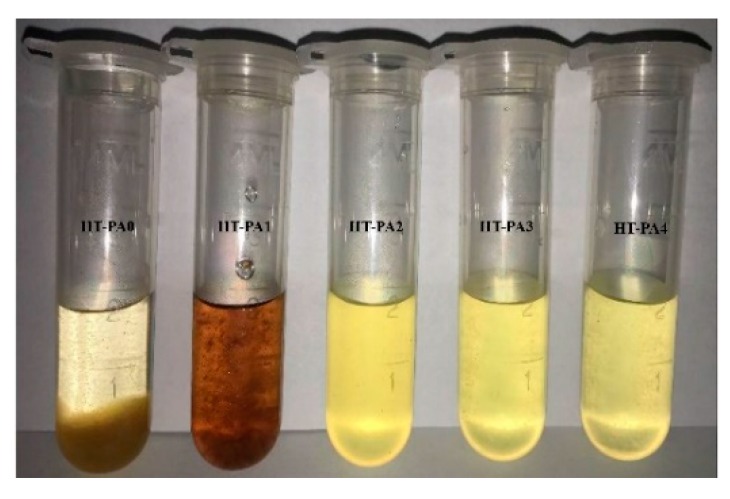
Digital photo of the solubility of polyamides in DMF solution. Reproduced from Reference [71].

Different triptycene-based polyamides and polyimides with good solubility have also been synthesized by Pinnau’s group [18,33,57]. However, these soluble microporous polymers are all polymer chains with non-network macromolecular structures. For the network PIMs, the solubility is not sufficient even though triptycene is incorporated in the backbone of the polymer. For example, Zhang et al. [63,64,65,66,67,68,69] modified triptycene to obtain triptycene derivatives with different kinds of active sites, and then the catalytic self-reaction between triptycene derivatives synthesized stone-like insoluble polymers. Furthermore, Ghanem et al. [40] selected triptycene with six reactive sites to synthesize the network polymer. Unfortunately, the trip(R)-PIMs obtained were all insoluble. The insolubility of network PIMs may result from the steric effects of an adjacent benzene ring [40,63,64,65,66,68,69,70], which restricts the polymer’s movement. Hence, in order to achieve soluble network PIMs, a contrastive study on the reactions between triptycene derivatives with aromatic dichlorides and alkyl dichlorides was conducted by our group [71] in order to investigate their solubility. Due to the high steric effect of the benzene ring, the polymers obtained by the reaction between triptycene derivatives and benzenedicarbonyl chlorides were not even soluble in aprotic polar solvents such as NMP. However, the reaction between triptycene derivatives and the alkyl dichloride resulted in a polymer with increased solubility in aprotic polar solvents due to the increase in length of the alkyl dichloride (Figure 15), which indicates that higher flexibility in the polymer chains would enhance the solubility under certain conditions.

## 6. Physical Aging Problem

**Figure 16:**
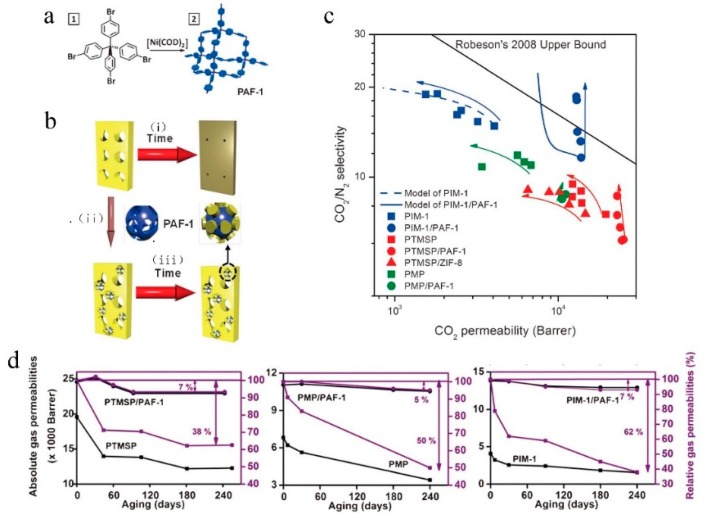
(**a**) Synthesis of PAF-1 particles. (**b**) Super glassy polymer/PAF-1 intermixing. Typically (i) PTMSP, PMP, and PIM-1 densify to give a non-permeable conformation, (ii) but with the addition of PAF-1, (iii) the original permeable structure is maintained. (**c**) Single gas CO_2_ permeability and ideal CO_2_/N_2_ selectivity of PTMSP (red), PMP (green), and PIM-1 (blue) based nanocomposites are plotted on Robeson Upper Bound. Te squares represent pure super glassy polymer films, circles represent super glassy polymer/PAF-1 films, and triangles represent super glassy polymer/metal–organic framework films. Our model accurately predicts the ging trends in different systems. Ultimately, PAF-1-based nanocomposites perform better over time without any CO_2_ permeability loss. The arrows provide a visual guide to show the relationship between CO_2_ permeability and selectivity over time (aging is tracked from the bottom of each arrow). (**d**) Absolute (black) and relative (purple) CO_2_ permeabilities of PTMSP-, PMP-, and PIM-1-based membranes, pristine polymers (squares), and polymer/PAF-1 (circles) membranes. Lines are drawn to guide the eye. The deviation of these permeability measurements is within ±10%. Purple arrows show the aging degree in these membranes. Adapted from Reference [90].

In nature, the formation of pores in a material is energetically unfavorable, which means that natural materials tend to be dense [85]. Theoretically, all porous materials will, thus, tend towards densification over time, where the packing density increases with the decrease in permeability. This phenomenon is especially severe if the selective layers are thin because of the increased mobility near the surface [3,23]. In order to retain the intrinsic pores in a material for as long as possible, a typical method is to use rigid monomers such as in the synthesis of inorganic zeolites [81]. However, the degree of rigidity in the monomer required for the control of physical aging in polymers remains unknown. Therefore, different rigid monomers have been used to suppress physical aging, and iptycene-based monomers are typical. Alghunaimi et al. [86] synthesized iptycene-based microporous polyimides and investigated their physical aging for the separation of CO_2_/CH_4_. These polymers exhibited minimal physical aging over 150 days, as shown by negligible changes in selectivities and small (15–20%) drops in permeability, which was attributed to the rigid and shape-persistent structure of iptycene due to its internal free volume. Pentiptycene is also used to resist against physical aging [87], and no obvious physical aging can be observed for pentiptycene-based polyimide films for separating gas pairs such as H_2_/N_2_, CO_2_/N_2_, etc. between aged (more than 130 days) films and fresh films. The excellent anti-physical aging results from the relatively “stable” free volume architecture because pentiptycene has built-in free volume, which are less likely to be susceptible to compaction due to chain relaxation. This result is also similar to our report [71] that a rigid 3D-contorted triptycene-based polymer membrane mitigated the physical aging because, on the one hand, triptycene has built-in free volume, which is already in an equilibrium state; On the other hand, the formed network structure using a triptycene monomer can restrict the mobility of the chains and form permeant micropores, which leads to good stability. Furthermore, if an all-rigid backbone was synthesized in polymers, its anti-physical aging would be excellent. Liang et al. [88] fabricated a conjugated microporous polymer membrane with a 3D network consisting of all-rigid conjugated skeletons, which provides excellent retention of solutes and high permeance. It also shows outstanding permanence stability by continuous filtration of PPh-IX (protoporphyrin-IX) in ethanol for 100 h. They ascribed this stability to the conjugated rigid skeleton in the polymer. Besides the incorporation of rigid monomers into the backbone to control physical aging, Lau et al. [89,90] tried to solve this problem by adding ultra-porous nanoparticles, porous aromatic frameworks(PAFs), in glassy polymers such PIM-1, PTMSP, etc. to form interwoven nanocomposites, which also show good stability because the regular carbonaceous pores in PAFs can harbor side chains of the glassy polymers and freeze the structures so that aging in glassy polymers was effectively inhibited. Therefore, CO_2_/N_2_ selectivity increased while CO_2_ permeability was reduced by less than 7% over the one-year investigation where the corresponding glassy polymer membranes showed reductions in CO_2_ permeability of 38% to 62% (Figure 16).

Meanwhile, as Bernardo et al. [23] reported in their systematic analysis of long-term aging in PIM-1, the alcohol-treated membrane was able to restore most of its original permeability. This renewed membrane exhibited slower physical aging than the original one. The reason for this may be the counteracting of the aging effect in thin films. Methanol soaking is often used to open pores in the microporous polymer membranes, including PIMs. The porous structures generated are in a non-equilibrium state, which lead to a faster densification over time as the polymer reaches its intrinsic equilibrium state. Therefore, if a membrane possesses micropores without requiring the methanol-soaking step and can be readily used for gas separation, its physical aging problem can be controlled well, even if it cannot be completely eliminated. In order to obtain such structures, the incorporation of rigid monomers with more than three active groups create a 3D network structure, which may be a suitable way to obtain a microporous polymer membrane with controllable physical aging.

## 7. Applications

The microporous polymer membranes (including PIMs) have exhibited extremely high separation performance in gas separation, and have been leveraged in polymer membranes to surpass the Robeson upper bounds, which open an entirely new methodology for gas separation [21,26]. Many reviews on gas separation using microporous polymer membranes have, thus, been published [4,5,16,21,91,92,93]. Besides excellent separation performance in gas separation, microporous polymer membranes can also exhibit good separation performance in other separations such as nanofiltration, pervaporation, and gas/vapor separation.

### 7.1. Nanofiltration

In addition to gas separation, nanofiltration, especially organic-solvent nanofiltration (OSN), may be another significant application of microporous polymer membranes because similar pore sizes are used in both separations, and because PIM polymers are soluble only in a few solvents such as tetrahydrofuran (THF) and chloroform. This suggests a great potential application in the OSN [94]. Furthermore, microporous polymers with a large free volume could display high diffusion coefficients and high flux. Eventually, high retention rates can be expected. Thin film composites (TFCs) made by blending of the PIM with polyethyleneimine were tested in OSN [94]. The results showed that, compared to the state-of-the-art industrial Starmem^TM^ 240 membranes and other poly(trimethylsilyl propyne) TFCs, better retention and much higher fluxes were achieved by the newly developed PIM TFC membranes, when tested in OSN with solvents such as n-heptane and toluene, as shown in Table 1.

**Figure 17:**
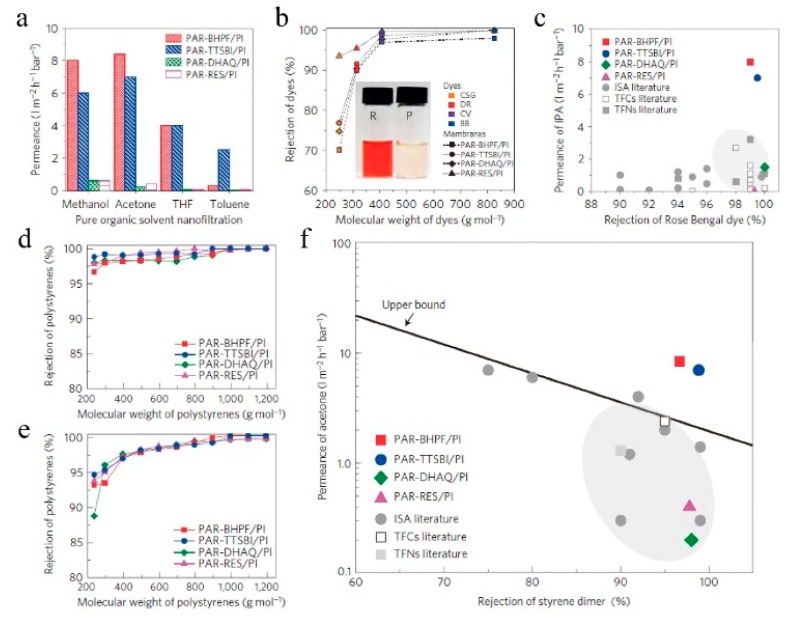
Organic solvent nanofiltration. (**a**) Pure solvent permeances for methanol, acetone, tetrahydrofuran (THF), and toluene through polyarylate (PAR) nanofilm composite membranes prepared on crosslinked polyimide (PI) supports. Nanofiltration was conducted in a crossflow filtration system at 30 °C under 30 bar. (**b**) Rejection versus molecular weight of dyes: brilliant blue (BB, 826 g/mol), crystal violet (CV, 408 g/mol), disperse red (DR, 314 g/mol), and chrysoidine G (CSG, 249 g/mol) in methanol. Inset photo shows the retentate (R, left) and permeate (P, right) samples for PAR-RES/PI. Nanofiltration was conducted separately for each dye in a crossflow filtration system at 30 °C under 30 bar. (**c**) Isopropanol (IPA) permeance versus the rejection of Rose Bengal (1017 g/mol) for polyarylate/PI nanofilm composite membranes versus typical, integrally skinned asymmetric (ISA), thin-film composite (TFC), and thin-film nanocomposite (TFN) membranes reported in the literature. Nanofiltration was conducted in a dead-end stirred cell (500 r.p.m.) at 30 °C under 30 bar. d and e) Rejection versus the molecular weight of polystyrene oligomers for polyarylate/PI nano-film composite membranes. Nanofiltration of a feed solution comprising polystyrene oligomers dissolved in acetone (**d**) or THF (**e**), respectively, which was conducted in a crossflow filtration system under 30 bar at 30 °C. (**f**) The permeance of acetone versus the rejection of α-methyl styrene dimer (236 g/mol) for polyarylate/PI nanofilm composite membranes. Typical nanofiltration data of ISA membranes, TFC membranes, and TFN membranes reported in the literature are included. Based on the reported literature value, the upper-bound line is manually added to show a trade-off between permeance of solvent and rejection of solute molecules. Reproduced from Reference [39].

However, these membranes are relatively thick and range from 300 to 800 nm. Yet, higher membrane thickness would generally lead to higher resistance, and, consequently, lower flux [95]. Livingston [96] postulated that ultrafast solvent permeance would be achieved when membrane thickness was reduced to 100 nm. Meanwhile, it was reported [85] that the thickness of microporous membranes should generally be higher than 20 nm to minimize undesirable flux resulting from non-selective defects and to maintain their high separation selectivity. In order to obtain ultrathin membranes, a network polymer should be employed instead of a linear polymer [39,96] because decreasing the thickness of the PIM-1 membrane below 100 nm resulted in a decrease rather than an increase in heptane permeance [97]. This abnormal phenomenon was ascribed to structural relaxation of the polymer molecules with non-network macromolecular structures in the thin membranes. Similar results have also been observed in gas permeation [98], which indicates that polymers with non-network macromolecular structures may not be suitable for ultrathin nanofilms [39,96]. Consequently, Livingston [39] reported the use of interfacial polymerization to synthesize a defect-free, highly cross-linked network of polyarylate nanofilms with a thickness as low as 20 nm. These membranes demonstrated outstanding separation performance in OSN (Figure 17). It was finally concluded that this work might inspire interfacial synthesis of a different family of microporous polymers, such as PIMs, to obtain ultrathin microporous membranes with great potential for applications in molecular separation, including organic solvent nanofiltration, gas separation, water purification, and desalination, and in hydrocarbon separations in the petrochemical industry. This further supported the fact that polymers with a network structure may be better than those with non-network macromolecular structures when used to prepare the membrane for separations.

**Figure 18:**
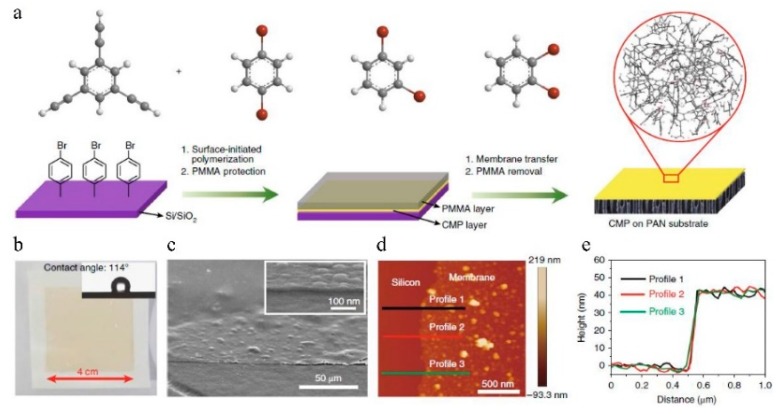
Preparation and characterization of all-conjugated CMP membranes with various dibromobenzene monomers, and investigation of their mechanical properties. (**a**) Surface-initiated polymerization of the CMP membrane on a bromobenzene-functionalized Si/SiO2 substrate. A Sonogashira–Hagihara reaction between 1,3,5-triethynylbenzene (1,3,5-TEB) and three different dibromobenzenes (whose structures are shown at the top) were adopted because it includes highly cross-linked and all-conjugated porous polymers. The bromine sites on the Si/SiO2 substrate (bottom left) acted as the initial reaction sites. The dibromo-monomers were used: 1,4-dibromobenzene, 1,3-dibromobenzene, and 1,2-dibromobenzene. Atoms color code: C, grey, H, white, and Br, red. The bottom panel shows a schematic representation of the surface-initiated polymerization. The inset in the red circle (right) shows the all-rigid skeleton of the resulting CMP membrane. (**b**) Photograph of an approximately 42 nm-thick p-CMP membrane transferred to a PAN substrate. The contact angle is 114° (inset), which indicates the hydrophobic nature of this membrane. (**c**) Large-area surface and cross-section (inset) SEM images of the CMP membrane after transferring it to a silicon substrate. (**d**,**e**) AFM image (**d**) and corresponding height profile (**e**) of a p-CMP membrane on top of a silicon wafer. A scratch was made to expose the wafer surface, which allowed for the measurement of the height from the silicon wafer surface to the upper membrane surface. Reproduced from Reference [88].

**Figure 19:**
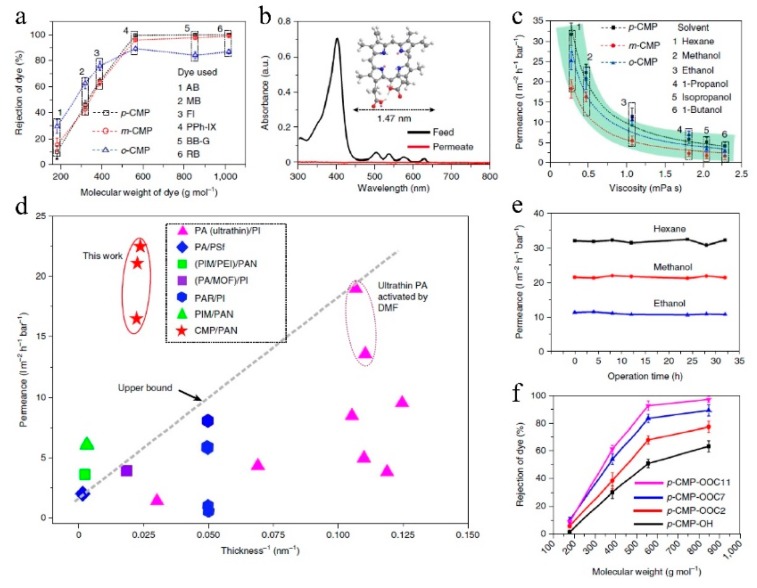
Nanofiltration performances of p-CMP, m-CMP, and o-CMP membranes. (**a**) Rejection behavior of different CMP membranes versus the molecular weight of various dyes (rose bengal (RB, 1017 g/mol), brilliant blue G (BB-G, 854 g/mol), PPh-IX (562 g/mol), fluorescein-4-isothiocyanate (FI, 389 g/mol), methylene blue (MB, 320 g/mol), and azobenzene (AB, 188 g/mol)) in ethanol. (**b**) Ultraviolet visible absorption spectra of PPh-IX dye in methanol to evaluate the separation performance of the p-CMP membrane. Inset: molecular structure of PPh-IX (C, grey, H, white, N, blue, O, red, lonepair electrons, pink). (**c**) Plot of solvent permeance through different CMP membranes against the solvent viscosity. The dashed curves show the reciprocal fitting of the permeance through p-CMP (black), m-CMP (red), and o-CMP (blue). The green shading shows an inverse relationship between permeance and viscosity. (**d**) Permeance for methanol versus the reciprocal membrane thickness for p-CMP, m-CMP, and o-CMP. Previously reported polymer-based OSN membranes are included for comparison and consist of polyamide (PA), PIM, MOF, and polyarylate (PAR), polyimide (PI), polysulfone (PSf), polyetherimide (PEI), and *N*,*N*′-dimethylformamide (DMF). The name of each membrane refers to its separating layer on the left and supports the substrate on the right. In two cases, the separating layer is a hybrid material: (PIM/PEI) and (PA/MOF). The dashed line shows the upper bond of the solvent permeability (permeance per unit thickness). (**e**) Plot of hexane, methanol, and ethanol permeances with time for p-CMP membranes. (**f**) Rejection behavior of post-modified p-CMP-OH membranes by different moieties. All nanofiltration experiments were conducted in a dead-end cell at 25 °C under 1 bar. The error bars in (**a**,**c**,**f**) represent the standard deviation, which was calculated based on an average of three membranes. Reproduced from Reference [88].

Liang et al. [88] employed conjugated microporous polymers (CMPs) composed of only chemically inert carbon–carbon and carbon–hydrogen bonds to fabricate OSN membranes by surface-initiated polymerization (Figure 18). Results showed that abundant, interconnected, and permanent micropores in a 3D network endowed this microporous membrane with very high permeance to a wide range of solvents and a distinct molecular weight cut-off of 560 g/mol (Figure 19). A comparison of the methanol permeance versus reciprocal thickness of different membranes, including CMPs, polyamide, PIMs [99], polyarylate, and others, showed that only two types of membranes with permanent pores (PIMs and CMPs) exhibited permeance values above the dashed line that indicates the intrinsic capability of membranes for solvent transport. Furthermore, a 3D network structure in the CMPs are expected to effectively prevent structural relaxation in various solvents so that permeance cannot decrease even when the membrane thickness is less than 100 nm. In addition, its high solvent resistance in various organic media and tunable pores sizes and surfaces facilitate the development of next-generation polymer-based OSN membranes via rational molecular design. Therefore, this work also supports the idea that the polymer with a network structure may be better for the membrane preparation especially in the OSN process. Furthermore, 3D network structures in the CMPs are expected to effectively prevent structural relaxation in various solvents, so that permeance could not decrease even when the membrane thickness is less than 100 nm. Its high solvent resistance in various organic media, and tune-able pore sizes and surfaces facilitate the development of next-generation polymer-based OSN membranes via rational molecular design. Therefore, this work also supports the idea that polymers with network structures may be better for membranes used in OSN.

### 7.2. Pervaporation

The use of the microporous polymer membranes in pervaporation has already been studied such as in the use of the PTMSP membrane for the pervaporative separation of an ethanol/water solution or a butanol solution [100,101]. Both high selectivity and high flux can be obtained due to the high free volume in the membrane. The first separation application of PIMs was in the pervaporative removal of phenol from an aqueous solution [9]. Separation factors of 16–18 were obtained at temperatures of 50 to 80 °C and a phenol feed concentration in the range of 1 to 5 wt%, which indicated the hydrophobic character of the polymer. Although the separation performance was similar to that obtained by a poly(dimethylsiloxane) (PDMS) membrane, these results were obtained by a membrane without optimization. Thus, it was thought that higher selectivities would be achievable through chemical and structural modification without sacrificing the high fluxes. Adymkanov et al. [102] systematically investigated the pervaporation of aliphatic alcohols, water, and a water-alcohol mixture through a PIM-1 membrane. High permeability was reported, and permeability decreased, according to their kinetic factors due to the reduction in the diffusion coefficient. High free volume would lead to negative activation energy, which is often observed in PTMSP or amorphous Teflon AF2400. This was also observed for a PIM-1 membrane. Meanwhile, no rapid drop in permeability over time was observed for the PIM-1 membrane, which also suggests that PIM-1 or derivatives may be promising as new potential pervaporation materials. Table 2 shows the comparison of different membranes in the separation of an ethanol-water solution. The table shows that the separation performances of the microporous polymer membranes (PIMs and PTMSP) are relatively higher than those of the other membranes because they possess relatively higher free volumes. The free volume in the polymers would also affect the transport properties and solubility in liquids [103,104]. From this result, it suggested that the low-efficiency solution-diffusion process in the dense polymer membrane impedes enhancements in separation performance [88].

### 7.3. Gas/Vapor Separation

**Figure 20:**
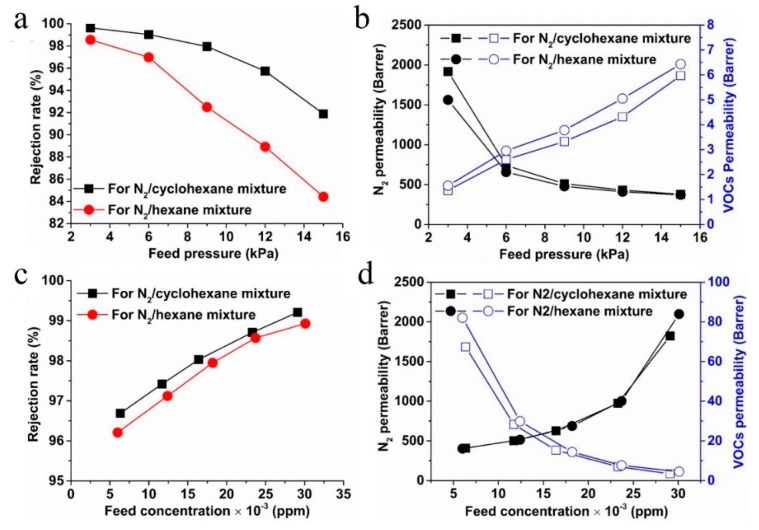
Effects of feed pressure (**a**,**b**), separation was conducted in a crossflow mode at 25 °C and about 30000 ± 1250 ppm feed VOC concentration, and feed concentration (**c**,**d**), separation was conducted in a crossflow mode at 25 °C under 4 kPa, on membrane separation performance. Reproduced from Reference [71].

As discussed above, microporous polymer membranes are widely used for gas separation and nanofiltration. There have been few reports on their use in gas/vapor separation, since some glassy polymer membranes may lose their selectivity in the presence of trace amounts of condensable compounds such as volatile organic compounds (VOCs) [110]. This would be of particular concern if the microporous polymer membrane were to be used for molecular sieving of N_2_ over VOCs because the affinities of VOCs for polymers are higher than that of N_2_ for polymers. Therefore, when the microporous membrane is used for gas/vapor separation. VOCs usually either permeate preferentially through the membrane (as in the case of PTMSP) or swell the membrane, which leads to low molecular sieving performance. However, if the pore size and the swelling can be controlled simultaneously, the molecular sieving separation performance can be excellent. In contrast, conventional microporous polymer materials have linear structures with good solution-processability, and may swell when exposed to VOCs. Therefore, polymers with network structures would be a good choice. In this context, we synthesized microporous polymers with network structures (Figure 10) using a triptycene derivative [71]. These unique polymers can only be dissolved in certain aprotic polar solvents such as dimethyl formamide (DMF) and show good swelling resistance in the presence of common VOCs such as cyclohexane, hexane, acetone etc., which make them an excellent membrane for molecular sieving of N_2_ over VOCs. Different N_2_/VOC mixtures and operating parameters have, thus, been employed to investigate membrane performance. Rejection rates as high as 99% and good permeability were obtained (Figure 20), and the larger molecules exhibited a higher rejection rate, which indicates that these unique membranes operate on a molecular sieving mechanism. Meanwhile, this membrane also showed good stability under certain conditions. It was concluded that the successful fabrication of soluble network PIMs membranes provides an important contribution to the fabrication and application of microporous polymer membranes.

## 8. Conclusions and Outlook

Polymers of intrinsic micro-porosity have been widely studied since their inception. Significant progresses have been achieved in the exploration of PIMs as membrane materials in different separation processes. Meanwhile, polymers with linear structures and network structures have also been synthesized. Polymers with a linear structure shows better mobility of the polymer chains, which leads to relatively good solubility in common solvents while the solubility of polymers with network structures is relatively inferior because most of such polymers exhibit stone-like solubility. However, polymers with network structures display better separation performance and are capable of forming nano-films with thicknesses less than 100 nm. Therefore, the development of polymers with network structures should be one of the main research directions for PIMs. Furthermore, although most PIMs membranes have been used for gas separation, there are a few papers describing their use in other separations such as nano-filtration, pervaporation, and gas/vapor separation. These successful applications indicate the excellent separation performance of PIMs membranes in a wide variety of separations, which suggests wide applicability of PIM membranes in the near future, especially considering the continual development of new polymer materials. Despite significant achievements in the development of PIMs, many challenges still remain in relation to the industrialization of PIMs as membrane materials, especially due to the physical aging problem. Although many methods have been developed in an attempt to rectify this issue, and many reviews have summarized the structure-aging relationship, no great breakthrough has been realized, and physical aging behavior that meets the stability requirement of industrial applications has still not been achieved. According to current studies, we believe that that the usage of rigid monomers such as triptycene or formation of a rigid network backbone in the polymer chains may be the most realistic way to control physical aging in the microporous polymers since monomers such as triptycene have built-in free volume and the rigid structures restrict a larger conformational change of the chains. This leads to the formation of permanent micropores in the polymers and, therefore, excellent anti-physical aging properties. Furthermore, Freeman et al. [12] pointed out in their recent review that investigating structure-property relationships may not be possible to yield breakthrough materials. It may only be possible to tailor membrane materials for desired separation performance and to improve the understanding of inherent correlations. Therefore, the development of new materials that overcome this challenge, such as PIMs with pure network structures, high rigidity, and selective solubility in certain solvents, is important for the industrialization of these polymers in the future. However, using more rigid monomers to synthesize microporous polymers would lead to a problem of insolubility, which further affect the film-forming properties. Therefore, the trade-off between stability and solubility resulting from the use of rigid monomers should be investigated in detail. Yet, in-situ polymerizations such as interfacial polymerization or surface-initiated polymerization are good methods to overcome this trade-off problem. Furthermore, mixed matrix materials such as blending of microporous nanoparticles in microporous polymers will also provide good gas separation performance since it can combine the advantages of particles of higher selectivities and microporous polymers of easy film-forming properties. Simultaneously, high aging resistance may also be obtained, as discussed above. However, due to the different properties, how to make them compatible or uniform in terms of the dispersion of particles in the polymers requires more attention. In addition, mixed matrix materials cannot be used for the formation of thin film composite membrane, especially when the size of particles is similar or greater than the thickness of the selective layer.

Furthermore, Van der Bruggen et al. [111] suggested that a real breakthrough of techniques should not only be expected from the preparation of new and improved membrane materials (although this is, indeed, very important in some applications and fields), but also from the smooth integration of membrane technology in the tool box of chemical engineers. This is because the actual priority in the industrialization is stable performance over time rather than achieving the highest separation performance in terms of selectivity and flux. Therefore, in future research, more attention should be paid to the problem of physical aging rather than solely focusing on achieving maximum separation performance in PIMs because the separation performance obtained by the use of recently synthesized polymers has already exceeded that of the membranes used industrially.

In summary, PIMs are promising materials with excellent potential applications. We also firmly believe that, with further developments in PIMs, PIMs will be one of the most likely candidate materials for industrialization.

## Figures and Tables

**Table 1:** Retention characteristics of various membranes, as measured by the retention of a polystyrene mix in n-heptane and toluene. Blend membranes with PEI were thermally crosslinked at 120 °C for 16 h. Reproduced from References [94].

No.	Polymer	Solvent	Pressure (bar)	90% Retention (g/mol)	99% Retention (g/mol)	Permeance (l/(m^2^hbar))	Permeance (kg/(m^2^hbar))	Time (h)
1	StarmemTM240	n-Heptane	30	~400	~1400	0.1	0.1	50
2	PIM-1	n-Heptane	4	560	-	7.3	5.0	3
3	PIM-1	n-Heptane	10	290	420	4.2	2.9	230
4	PIM-1	n-Heptane	20	240	400	4.1	2.8	250
5	PIM-1	n-Heptane	30	<200	410	4.0	2.8	280
6	StarmemTM240	Toluene	30	380	1200	0.7	0.6	140
7	PIM-1/20%PEI	Toluene	10	480	890	3.2	2.8	>200
8	PIM-1/20%PEI	Toluene	20	430	790	3.1	2.7	>200
9	PIM-1/20%PEI	Toluene	30	460	860	3.1	2.7	>200
10	PIM-1-CO1-50/20%PEI	Toluene	10	350	680	0.9	0.8	>200
11	PIM-1-CO1-50/20%PEI	Toluene	20	330	680	0.9	0.8	>200
12	PIM-1-CO1-50/20%PEI	Toluene	30	340	680	0.9	0.8	>200

**Table 2:** Comparisons of the separation performance for the pervaporative separation of ethanol-water solution for different polymer membranes.

Membrane	Feed Ethanol Concentration, wt%	T, ℃	Separation Factor	Flux, Kg/m^2^h	Reference
PIM	10	30	10.7	0.47	[102]
	10	50	10.2	1.10	
PDMS	5	70	7.6	1.667	[105]
PDMS-PAN membrane	6	30	8	3.5	[106]
PTMSP	10	50	12	3.5	[101]
Pervap4060	10	50	7	1.9	[101]
Pevatech PDMS	10	50	6	3.3	[101]
SBR	10	30	3.8	0.015	[107]
poly (styrene-co-utylacrylate) copolymer	5	30	16	0.3	[108]
TMVS-g-PVDF	10	30	~3.1	2.85	[109]

Styrene Butadiene Rubber (SBR). Poly Dimethyl Siloxane (PDMS). Polyacrylonitrile (PAN).
